# SPARK: an mHealth intervention for self-management and treatment of gestational diabetes mellitus in Sweden – protocol for a randomised controlled trial

**DOI:** 10.1136/bmjopen-2024-089355

**Published:** 2025-03-03

**Authors:** Caroline Lilliecreutz, Emmie Söderström, Matilda Ersson, Marcus Bendtsen, Victoria Brown, Nina Kaegi-Braun, Rebecka Linder, Ralph Maddison, Simona I Chisalita, Marie Löf

**Affiliations:** 1 Department of Biomedical and Clinical Sciences, Linköping University, Linkoping, Sweden; 2 Department of Medicine, Huddinge, Karolinska Institutet, Stockholm, Sweden; 3 Department of Health, Medicine and Caring Sciences, Linköping University, Linkoping, Sweden; 4 Deakin University, Melbourne, Victoria, Australia; 5 Endocrinology Clinic, Linköping University Hospital, Linkoping, Sweden

**Keywords:** Diabetes in pregnancy, Randomized Controlled Trial, Behavior, Maternal medicine, Cardiovascular Disease, eHealth

## Abstract

**ABSTRACT:**

**Introduction:**

Gestational diabetes mellitus (GDM) is increasingly becoming a serious public health challenge. Innovative, effective and scalable lifestyle interventions to support women with GDM to manage their condition and prevent adverse obstetric and neonatal outcomes as well as later morbidity are required. This study aims to evaluate whether a novel, multilingual and scalable mobile health (mHealth) intervention (SPARK; SmartPhone App for gestational diabetes patients suppoRting Key lifestyle behaviours and glucose control) can improve self-management and treatment of GDM and prevent adverse maternal and offspring outcomes.

**Methods/analyses:**

SPARK is a multicentre two-arm randomised controlled trial recruiting women diagnosed with GDM in south-eastern Sweden. A total of 412 women will be randomised to either standard care (control) or the SPARK intervention. The SPARK online platform (accessed through a mobile app) provides a behaviour change programme for healthy eating, physical activity and glycaemic control. To increase reach, SPARK is available in Swedish, English, Arabic and Somali. SPARK also comes with a clinician portal where healthcare professionals monitor and intervene when glycaemic control is unsatisfactory (above certain cut-offs). Primary outcomes are glycaemic control that is, time in range and HbA1c, while diet, physical activity (ActiGraph), gestational weight gain, metabolic and inflammatory biomarkers in weeks 37–38, adherence to protocol for daily glucose sampling, as well as adverse obstetric and neonatal outcomes are secondary outcomes. Secondary outcomes also include cardiometabolic risk evaluation, physical activity and healthy eating behaviours 1 year postpartum. A health economic evaluation of SPARK vs standard care will also be conducted.

**Ethics and dissemination:**

This study has been approved by the Swedish Ethical Review Authority (2021-06627-01; 2022-03842-02; 2023-05911-02). Results will be disseminated through scientific papers in peer-reviewed journals, posts in traditional and social media, and presentations at scientific and healthcare professionals’ conferences.

**Trial registration number:**

This trial was registered at the ClinicalTrials.gov register platform (ID NCT05348863) 27 April 2022.

**STRENGTHS AND LIMITATIONS OF THIS STUDY**

- The SPARK intervention includes a lifestyle behaviour change programme for women with gestational diabetes mellitus and a clinician portal where healthcare professionals monitor and intervene when glycaemic control is unsatisfactory (based on novel algorithms).
- Strengths of this randomised controlled trial comparing the SPARK intervention to standard care are that it includes a cost-effectiveness analysis and a process evaluation. Moreover, the intervention also includes a postpartum support programme.
- Another key and novel strength is that the behaviour change programme is accessible in Swedish, English, Somali and Arabic to increase reach of the intervention.
- There may be some women who do not speak any of the four languages or are illiterate and thus cannot be enrolled; however, this limitation is minor as the main languages in this population are covered, and key intervention content is also summarised and available in audio files to enable access for women with limited reading skills.

## Introduction

 Gestational diabetes mellitus (GDM), defined as a glucose intolerance disorder with onset during pregnancy,^1^ affects one out of six pregnancies globally,^2^ and is currently the most common medical complication of pregnancy.^3^ The prevalence of GDM has increased due to higher maternal age and increased obesity rates as well as new stricter WHO diagnostic criteria from 2013.^4^ GDM is associated with adverse obstetric and neonatal outcomes such as pre-eclampsia, large-for-gestational-age infants, caesarean section and shoulder dystocia.^5 6^ Furthermore, women with GDM are 50%–80% more likely to develop GDM in subsequent pregnancies^6^,^8^ and have higher risks for type 2 diabetes, cardiovascular morbidity^9^,^11^ and mortality^12^ as well as offspring impaired glucose tolerance.^2 10^

Given the serious maternal and foetal consequences, GDM requires careful management during pregnancy. The primary treatment of GDM includes dietary and physical activity modifications as well as daily self-monitoring of plasma glucose.^13^ Indeed, diet and physical activity modifications are sufficient to control glycaemic status in 70%–85% of women with GDM^3^ and have proven effective in decreasing maternal and foetal morbidity.^14 15^ However, this treatment is primarily provided using traditional education approaches and face-to-face consultation. Such procedures are limited by cost, lack of resources (personnel and physical location) and inability to provide broad coverage.^16^ Also, qualitative research has shown that women with GDM express an immediate need of information about their diagnosis and consequences,^17^ which may be difficult to provide when relying on face-to-face education. Currently, the management of GDM is particularly challenging due to the increasing GDM prevalence, making it difficult for clinics to provide sufficient treatment for all women diagnosed with GDM. Clearly, effective and scalable solutions for self-management of GDM with minimal supervision from healthcare professionals are required to meet the demand. Preferably, such solutions should incorporate long-term support for women with GDM to prevent type 2 diabetes and cardiovascular events in the first years postpartum, as they are lacking but requested.^18^

Digital technologies are becoming important resources for healthcare services. Mobile health (mHealth) refers to the use of mobile and wireless devices (mobile phones, tablets, wearable sensors etc) with advantages such as real-time monitoring and feedback, less burden on healthcare systems as well as cost-effectiveness.^19^ mHealth has been successfully integrated into interventions to promote healthy diets, physical activity and weight loss.^20 21^ We have previously shown that a smart phone intervention (HealthyMoms) successfully improved eating habits and lowered gestational weight gain in healthy women during pregnancy.^22^ Given the positive effect of the app, it has the potential to be adapted and expanded to reach other pregnant populations as well, including women diagnosed with GDM. Recently, trials of mHealth interventions targeting GDM patients have shown promising results regarding compliance and glycaemic control,^23^,^27^ and women as well as healthcare professionals have expressed the potential of such tools.^28 29^ However, some of them were conducted in countries with healthcare systems that are not comparable to Swedish GDM care.^23 24 27 30^ Thus, even though principles for GDM management are similar to other countries and follow international guidelines with glucose monitoring, dietary and physical activity advice with escalation into pharmaceutical treatment (metformin, insulin) when required,^4^ there are differences in how primary and secondary care are organised and executed, which motivates mHealth interventions that are adapted and evaluated in the Swedish healthcare system. Furthermore, some of the previous interventions were used in addition to usual care rather than substituting physical healthcare visits,^24^,^27^ while other trials investigated a remote glucose management intervention without additional lifestyle interventions.^23^ Also, economic evaluation of mHealth interventions is largely lacking.^31^ Indeed, in the Swedish healthcare context, a high-quality trial investigating a holistic, theory-guided mHealth intervention for women with GDM is lacking. Furthermore, previous research has used HbA1c (glycosylated haemoglobin) as an outcome for glycaemic control. However, HbA1c only reflects an average measure for the preceding 8–10 weeks and may be a suboptimal measure of foetal glycaemic exposure since it can be falsely low due to increased erythropoiesis during pregnancy.^32^ Finally, migration continues to increase worldwide and many countries such as Sweden are increasingly becoming more ethnically and culturally diverse. It is well-known that GDM is more prevalent among non-European populations,^33^ and recent reports also indicate that this applies to Sweden in both first and second generation immigrants.^34 35^ Adaptations and tailoring for foreign-born women who do not speak Swedish are thus essential to enable interventions to become accessible and inclusive for all women.^36^

This protocol describes a multicentre randomised controlled trial, with the overall aim to evaluate a scalable and multilingual (Swedish, English, Somali and Arabic) mHealth intervention called SPARK (SmartPhone App for gestational diabetes patients suppoRting Key lifestyle behaviours and glucose control) in terms of self-management and treatment of GDM, prevention of adverse obstetric and neonatal outcomes as well as improvement of cardiometabolic health in women diagnosed with GDM. The specific aims of the trial are to compare the SPARK intervention vs standard care regarding the following aspects:

(a) glycaemic control in the third trimester (primary outcome); (b) gestational weight gain, moderate-to-vigorous physical activity, eating behaviours and metabolic and inflammatory biomarkers in the third trimester; (c) adherence to daily blood glucose sampling protocol; (d) adverse obstetric and neonatal outcomes; and (e) cardiometabolic risk, physical activity and eating behaviours 1 year postpartum. We will also evaluate cost-effectiveness and the potential translation of SPARK into real-world healthcare settings.

## Methods and analysis

### Trial design overview

**Figure 1:**
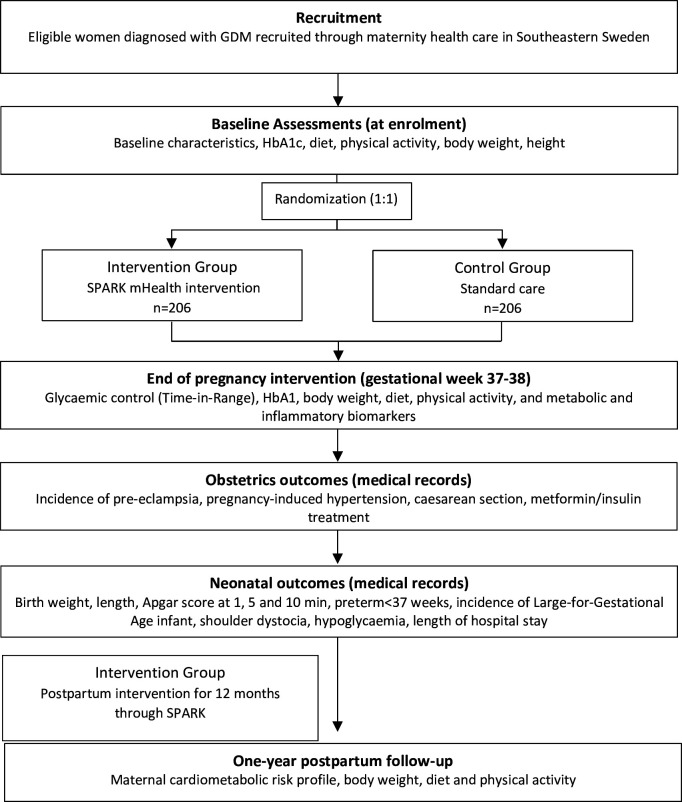
Study outline for the SPARK randomised controlled trial.

The SPARK trial is a two-arm parallel group multicentre randomised controlled trial. Following baseline assessment, women diagnosed with GDM will be randomised to the intervention group (SPARK mHealth intervention) or the control group (standard care). Outcomes are assessed in gestational weeks 37–38, at delivery and 1 year postpartum (figure 1). This protocol follows the SPIRIT statement^37 38^ (online supplemental material file 1), and the intervention results will be reported in accordance with the CONSORT guidelines^39^ with additional components for eHealth/mHealth research.^40^ We do not foresee any protocol amendments, but if they occur, they will be reported in the trial registration.

### Intervention condition

**Figure 2:**
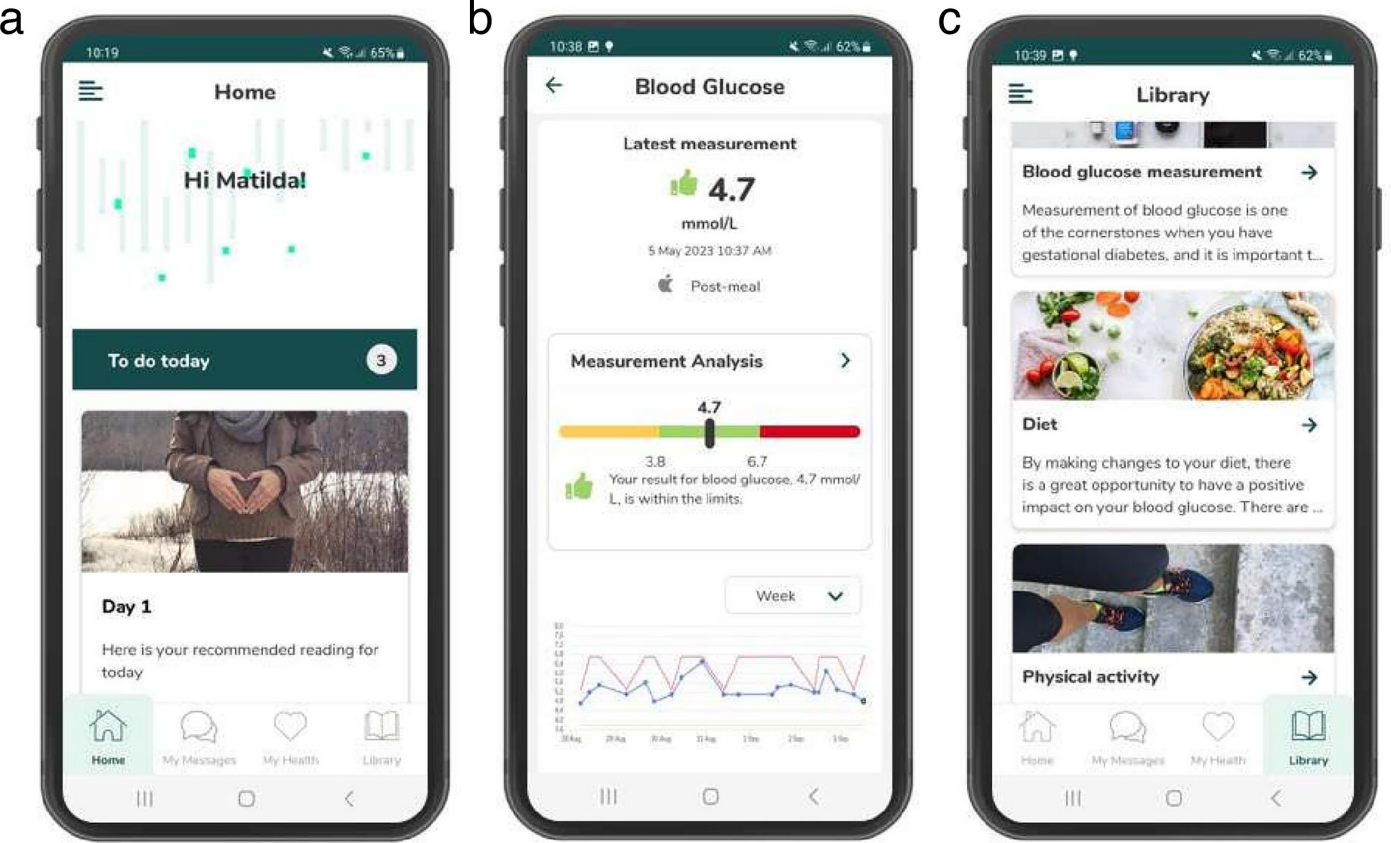
The SPARK user interface (app) for the patients showing examples from (a) the home (starting) page; (**b**) blood glucose monitoring using a glucometer automatically transferred via Bluetooth with instant feedback and graphs visualising values over time; and (c) interventional content to support healthy eating and physical activity.

Overall: The SPARK mHealth intervention will be delivered via an online platform (Cuviva@) and includes the following: (a) a mobile user interface for patients (the SPARK app, for iOS or Android) for automatic uploading of blood glucose values with instant feedback and a behaviour change programme to improve dietary and physical activity behaviours during pregnancy (figure 2); (b) a clinician user interface for desktop where healthcare professionals can monitor blood glucose values and interact with their patients during pregnancy; and (c) a 12-month postpartum programme to promote healthy eating, physical activity and healthy weight after delivery.

Development: SPARK is built on our previous HealthyMoms1.0 technical platform with content promoting healthy eating, recommended gestational weight gain and physical activity in pregnant women.^22^ The development of new content for SPARK was based on formative work, including interviews with healthcare professionals (eg, midwives, endocrinologists, dieticians, obstetricians) working with GDM patients and end-users (unpublished data). The content was developed by a team with extensive experience in nutrition, GDM, physical activity, behaviour change and obstetrics. GDM patients and healthcare professionals provided feedback on content and design throughout the development process.

Content: The SPARK app provides a comprehensive behaviour change programme to improve dietary and physical activity behaviours, based on current guidelines (www.slv.se). The programme is grounded in social cognitive theory and key behavioural change techniques^41 42^ including shaping knowledge, goal setting, identification of barriers, self-monitoring and feedback, which are important for promoting a healthy lifestyle.^43^ It consists of the following features: an education programme on healthy eating behaviours, foods and drinks to manage GDM; an exercise guide (eg, aerobic and resistance exercises and training programmes); and self-monitoring with feedback. The self-monitoring feature includes the following variables: daily intakes of fruits and vegetables, sweetened beverages, juice, and milk, number of meals, walks after meals, and time spent on moderate and vigorous physical activity. Results are shown graphically to provide feedback and comparison to relevant guidelines. The app also reminds women to measure their blood glucose values using a glucometer four times per day (fasting and three postprandial values) and enables automatic data transfer via Bluetooth to SPARK. Women will also receive instant feedback on whether their uploaded value is within or outside the recommended range. Participants moreover receive automated push notifications two times per day to encourage them to keep using the app. Healthcare professionals (a midwife or dietitian with support from a consulting endocrinologist) monitor their patients via the platform, and patients are sorted to facilitate clinical decision making. Patients with glucose levels outside the treatment target, that is, three values above range or one value below range in the last 7 days (ie, unsatisfactory levels) are flagged and prioritised in the system. Support, communication and information about additional treatments (ie, metformin or insulin) will be managed through SPARK’s secure chat function only. In contrast, for patients with satisfactory values, messages are sent that encourage the patient to continue with their behaviours. After delivery, the app content is adapted to dietary and physical activity recommendations for the postpartum period based on national current guidelines (www.slv.se). This includes the possibility to self-monitor body weight (in relation to pre-pregnancy weight or other self-set goal by the patient), as well as the self-monitoring of daily intakes of fruits and vegetables, sweetened beverages, walks and pelvic floor training which are all graphically visualised to show trends and compared with guidelines when relevant. This content is available for 12 months postpartum.

Accessibility: Since many women diagnosed with GDM in Sweden are foreign-born and do not speak Swedish, the SPARK app has been translated and tailored to the most common languages for this target group (ie, English, Arabic and Somali) to be accessible and inclusive in accordance with our ongoing research in child healthcare.^44^

Data management: When a woman has taken her blood glucose using a glucose metre, the value is transferred via Bluetooth to the SPARK app. The woman is asked to press a button called ‘Save’ and data is then transferred to a cloud service located in Sweden and at the same time shown in the SPARK app and in the healthcare professionals user interface. No data is stored on the woman’s smartphone, so every time she uses the app, the data is collected from the cloud service. The procedure was approved by healthcare before study initiation and follows current legislations for our country.

### Control condition

The women in the control group will receive standard care, which includes (a) a standard ‘one-size-fits- all’ lecture on healthy diet and physical activity for GDM; (b) information regarding how to take and record their blood glucose values in an app called Glooko four times a day; and (c) a weekly review of their glucose values reviewed by a clinician (dietician or midwife with support from a consulting endocrinologist if required) followed by a phone call or email if deemed necessary, with contact decreased even further for patients with satisfactory response to treatment and without any pregnancy complications in accordance with local guidelines. Glooko is used for standard diabetes care in Sweden with the recorded blood glucose levels being stored in a cloud service approved by healthcare. The Glooko app is only used to upload glucose levels.

### Inclusion and exclusion criteria

Eligible pregnant women will be enrolled via maternity healthcare centres in south-eastern Sweden. Inclusion criteria are as follows: confirmed diagnosis of GDM detected by an oral glucose tolerance test in accordance to local guidelines based on the WHO diagnostic criteria from 2013,^4 45^ and ability to read and speak Swedish, English, Arabic or Somali sufficiently well to be able to provide informed consent. Also, key intervention content is also summarised in audio-files, to facilitate for women with limited reading skills. Exclusion criteria include known pre-pregnancy diabetes, twin pregnancy, age below 18, severe comorbidities that would limit participation or a previously diagnosed severe psychiatric illness.

### Recruitment

Study information is provided by assistant nurses to potential participants after they have been diagnosed with GDM at the maternity healthcare centres. Women who express interest in participating in the trial are contacted by the research team for additional information and the opportunity to ask questions before providing informed consent (online supplemental material file 2). All participants download the SPARK app on their smartphone and are asked to fill in a baseline questionnaire within the app. The control group only use a basic version of the app for filling in baseline and follow-up questionnaires while the intervention group also receives the intervention through the app as described above.

### Randomisation and blinding

After baseline measurements have been completed, participants are randomised in a 1:1 ratio to either the intervention (SPARK mHealth intervention) or the control group (standard care). Randomisation will be based on restricted randomisation, generated through STATA and administered by study personnel through sealed envelopes to preserve allocation concealment. Due to the design of the study with a digital intervention, participants and healthcare personnel are not blinded to their group allocation.

### Measures

#### Clinical outcomes

- Glycaemic control (time in range, TIR, % of time) in gestational weeks 37–38*.
- HbA1c in gestational weeks 37–38*.
- Metabolic and inflammatory biomarkers in gestational weeks 37–38.
- Gestational weight gain.
- Metformin/insulin treatment (introduction of).
- Adverse obstetric and neonatal outcomes (for details, see below).
- Maternal cardiometabolic risk profile 1 year postpartum.

#### Behavioural outcomes

- Dietary habits in gestational weeks 37–38 and 1 year postpartum.
- Physical activity in gestational weeks 37–38 and 1 year postpartum.
- Adherence to the measurement protocol for daily glucose samples during the first 2 weeks and the full intervention period.

Outcomes marked with * are the primary outcomes of this trial.

#### Glycaemic control

The primary outcome is glycaemic control (time in range, TIR, % of time according to recent international clinical target levels^13^ with local guidelines taken into consideration assessed using the blinded FreeStyle Libre continuous glucose monitoring (CGM) system (https://www.freestyle.abbott/us-en/products/freestyle-14-day.html), which via a subcutaneous sensor on the back of the upper arm measures interstitial fluid glucose levels every 15th minute for 14 consecutive days. For long-term glycaemic control and to be able to compare the trial effect with previous face-to-face interventions, we will also evaluate glucose homeostasis as HbA1c in gestational weeks 37–38 (assessed in routine care) as primary outcome (in accordance with trial registration). To complement these primary outcomes for glycaemic control, we will also analyse metrics of glycaemic variability such as mean average glucose excursion from the CGM system over 24 hours,^13^ and capillary sampling (fasting and postprandial values) in relation to target blood glucose levels during the full intervention period. Compliance to the instructions for self-monitoring of capillary blood glucose values will also be compared between the intervention and control group ((number of recordings/number of recordings according to the instructions) × 100)) based on the daily glucose metre data during the full intervention period. Altogether, these variables, although listed as secondary outcomes, will provide comprehensive data on when glycaemic control was reached and used to support interpretation of the results of the two primary outcomes.

#### Diet and physical activity

Intake of foods and drinks will be assessed through data collected by the web-based dietary recall method Riksmaten FLEX developed by the Swedish National Food Agency^46^ and adapted for pregnant women.^22^ We will calculate diet quality using the Swedish Healthy Eating Index score based on intakes of fruit and vegetables (g/day), fish and shellfish (g/day), red meat (g/week), fibre (g/10 MJ), wholegrain (g/MJ), polyunsaturated fat (E%), monounsaturated fat (E%), saturated fat (E%) and sucrose (E%) as previously described,^22 47^ with potential modifications considering the new Swedish food-based dietary guidelines (to be released in 2024). Women also complete the Food Insecurity Experience Scale Survey Module (FIES-SM) which consists of eight questions regarding access to adequate food.^48^ Questions focus on self-reported food-related behaviours and experiences associated with increasing difficulties in accessing food due to resource constraints. Physical activity and sedentary behaviour are assessed over eight consecutive 24-hour periods with the wrist-worn triaxial accelerometer ActiGraph GT3X (www.actigraphcorp.com). During these 8 days, participants record their waking hours and an activity questionnaire. Recording, data processing and conversion of the recorded movements into time spent in different activity levels including moderate-to-vigorous physical activity using appropriate cut-offs will be conducted as we recently described.^22^

#### Weight

Weight is measured using a standard procedure, where all women are measured wearing light clothing. Gestational weight gain will be calculated as measured weight in gestational weeks 37–38 minus the weight at baseline and will be adjusted for number of weeks between measurements. Weight gain will be classified as being within, below or above the recommendations for gestational weight gain, which has been provided by the National Academy of Medicine.^49^

#### Adverse obstetric and neonatal outcomes

Information about obstetric and neonatal outcomes are collected as continuous variables or incidence from electronic medical records including its registry (Obstetrix, ORACLE Cerner, Sweden). They include, for instance, the incidence of pre-eclampsia, pregnancy-induced hypertension, metformin/insulin treatment, gestational week at birth, induction of labour, caesarean sections, vacuum-assisted delivery, epidural anaesthesia, glucose-insulin treatment during delivery, infant Apgar score at 1, 5 and 10 min, incidence of large-for-gestational-age infants, macrosomia (>4500 grams), infant shoulder dystocia, infant hypoglycaemia and length of hospital stay. We will also evaluate infant birth weight and length as continuous variables as outcomes.

#### Metabolic and inflammatory biomarkers

Serum samples are taken, centrifuged and stored at −70°C at Linköping University’s Biobank Facility. They will be analysed for insulin-like growth factor I, and its binding proteins I and II (immunoenzymometric assay kit, Medix Biochemica, Finland, and ELISA kit, My Biosource, San Diego, USA, respectively), copeptin, midregional pro-atrial natriuretic peptide (MR-proADM) and midregional pro-adrenomedullin (MR-proANP) (time-resolved amplified cryptate emission technology assay, Brahms Kryptor, Henningsdorf, Germany) and leptin (Milliplex MAP Gut hormone panel, Merck Millipore, MA, USA).^50^,^54^ These biomarkers are selected as they have been linked to the development of infant macrosomia, pre-eclampsia and infant growth restriction, as well as the postpartum development of type 2 diabetes or cardiovascular morbidity and mortality^5153^,^56^; however, other biomarkers may also be considered due to new knowledge at the time of the analyses.

#### Maternal Cardiometabolic risk profile one year postpartum

A fasting blood sample is taken and plasma glucose, HDL-cholesterol, and triglycerides, serum insulin and insulin resistance (HOMA-IR) are assessed at the Department of Clinical Chemistry, Linköping University Hospital, as previously described.^22^ Waist circumference is measured at the umbilical location with a tape measure at the end of a normal expiration. The systolic and diastolic blood pressure are measured in a sitting position using an electric sphygmomanometer (ProBP 3400 series, WelchAllyn, NY, USA) with standardised procedures. Thereafter, based on these variables, a cardiometabolic risk score for each woman will be calculated using commonly applied standards.^57 58^ Also, to evaluate the microvascular function in women with previous GDM, a forearm skin comprehensive microcirculatory assessment will be performed using the PeriFlux PF6000 EPOS (Enhanced Perfusion and Oxygen Saturation) system measuring oxygen saturation and total speed perfusion. The collection of data in absolute units facilitates comparison of data between individuals, not possible with traditional laser Doppler techniques.^59^ Impaired microvascular dysfunction was recently identified in women with previous pre-eclampsia,^60^ and it has also been shown to be associated with increased cardiovascular risk profiles such as a higher SCORE2.^61^

#### Economic evaluation

A within-trial cost-effectiveness analysis^61^ in 2025 Swedish Krona will be conducted from a public healthcare system perspective, comparing the intervention to a standard care comparator. Costs will include intervention costs, other healthcare costs (eg, telephone calls, hospital visits) and will be estimated using unit costing principles and trial records, administrative data and published sources. Health-related quality of life will be measured using commonly applied questionnaires (AQoL-4D) (http://aqol.com.au/index.php/aqolinstruments) and incremental cost-effectiveness ratios will be estimated (including cost per quality-adjusted life year (QALY) gained, cost per percentage increase in glycaemic control, cost per unit improvement in Swedish Healthy Eating Index score, costs per unit improvement in maternal cardiometabolic risk factors). Non-parametric bootstrapping using 1000 samples will characterise uncertainty, and results will be presented on cost-effectiveness planes. Sensitivity analyses will moreover explore the robustness of results when varying key input parameters. For estimation of long-term consequences, beyond the trial period, decision analytical modelling will extrapolate, for example, cardiometabolic risk outcomes using Markov modelling, and employing trial data with evidence from external sources.^61 62^

#### Process and Scalability evaluation

At the end of the intervention (gestational weeks 37–38 and 1 year postpartum), women in the intervention group complete a questionnaire on their overall satisfaction with the app, namely their use and perceptions in terms of usability, design, and features in accordance with our previous studies.^22 44^ Correspondingly, semistructured interviews (audio) are conducted within a subsample of women in the intervention group (n=15–20 end of pregnancy as well as 1 year postpartum) and healthcare professionals (n=10–20) with different roles in the GDM treatment process (midwife, endocrinologist, dietician) to gain additional insight into their perceptions of the SPARK app and clinician portal and its potential for implementation at scale, including barriers and facilitators for such an implementation. All interviews will be transcribed and analysed using thematic analysis.^63^

### Statistical analyses

All analyses will include participants in the groups to which they were randomised (intention-to-treat). We will conduct both available data analyses and analyses with data imputed using multiple imputations with chained equations^64^ (using 200 imputed datasets generated using 30 iterations). We will use multilevel regression with adaptive intercepts for clinics and estimate models using Bayesian inference.^65^ All intercepts and covariates will be given Student’s t priors, and dispersion parameters will be given half-Student’s t priors. The priors will be centred at 0 with a scale of 2.5 and 3 degrees of freedom. For reporting purposes, we will use medians of posterior distributions as point estimates of effects together with 95% compatibility intervals represented by the 2.5 and 97.5 percentiles of the posterior distributions.

For each outcome, we will calculate the posterior probability of two cases:

1. The posterior probability that the estimated effect favours the intervention group.
2. The lower and upper bound of effect estimates symmetrically around the null which most closely includes 95% of the posterior distribution.

The first case is a straightforward contrast of outcomes between groups and concerns the posterior probability of one group having better outcomes than the other (note that the posterior probability that the estimated effect favours the control group is one minus the posterior probability of the estimated effect favouring the intervention group). The second case concerns the posterior probability of the two groups having similar outcomes. We will identify which range of effects, symmetrically around the null, include 95% of the posterior distribution. For instance, for the main outcome TIR, we will identify the OR range (eg, 1/1.2 and 1.2 ORs) which most closely includes 95% of the posterior distribution.

This contrast has implications for implementation decisions as the two treatments contrasted (SPARK; standard care) have different costs and resource requirements associated with them.

#### Models

For the primary outcome TIR, we will use beta regression and report on the estimated OR with respect to group allocation. We will use linear regression for all other outcomes apart from the introduction of metformin/insulin treatment, adverse obstetric and neonatal outcomes, and adherence to the measurement protocol for daily glucose. The introduction of metformin/insulin treatment will be modelled using ordinal regression (no treatment<metformin<insulin), adverse outcomes will be modelled using negative binomial regression (counting the number of incidences adverse outcomes), and average measurement protocol adherence will be estimated using beta regression. Models will be adjusted for gestational week at baseline, age, pre-pregnancy Body Mass Index (BMI) (underweight and normal weight vs overweight and obese), previous GDM diagnosis, baseline HbA1c, as well as baseline measures of each respective outcome where available. For models of gestational weight gain, we will, in accordance with our previous trials,^22^ fit follow-up weight in gestational weeks 37–38 on group allocation and adjust for baseline weight as it is more robust against regression toward the mean. When relevant we will also additionally adjust for educational attainment (university degree vs no university degree).

#### Ancillary analyses

We will study effect modification of the intervention on primary and secondary outcomes with respect to baseline variables. This will be done by estimating regression models with interaction terms between group allocation and baseline variables (one model per variable and outcome). The baseline variables of interest are the following: pre-pregnancy BMI (underweight and normal weight vs overweight and obese), parity (0 vs ≥1), educational attainment (university degree vs no university degree) and country of birth (foreign-born vs Swedish-born).

#### Attrition analyses

We will investigate if there is systematic attrition by estimating logistic regression models with response/no-response as outcome. These models will include baseline variables as covariates and interaction terms between baseline variables and group allocation. We will use shrinkage priors to account for the excessive number of parameters in these models.

### Sample size

We conducted a Monte Carlo study to estimate the number of participants we need to recruit. We simulated scenarios where the difference in TIR between groups at follow-up was five percentage points, which is of clinical relevance in diabetic women to lower the risk of adverse pregnancy outcomes.^66^ The differences between groups that we simulated started from 70% TIR in the control group and 75% in the intervention group to 90% TIR in the control group and 95% in the intervention group. We used an SD of 12 percentage points in both groups in all simulations. Aiming for a power of 0.8 at the 0.05 significance level, we found that with 360 women completing follow-up we will have enough power to detect a statistically significant effect in all the scenarios simulated. Based on our previous interventions,^22^ we expect a dropout rate of 10%–15% and will therefore recruit 412 participants. It should be noted that our analyses follow a Bayesian paradigm where null-hypothesis testing is not the basis for scientific inference; thus, a predetermined sample size to protect against type I and II errors is not required. Therefore, the function of our power calculation is only to give a target for recruitment.

### Adverse outcomes

The SPARK mHealth intervention was developed in close collaboration with healthcare professionals working with GDM treatment and in accordance with current clinical guidelines. Thus, we consider the potential risks for any harmful events with the intervention as minimal. It is also relevant to note that, if any such events should still occur, since the women are carefully monitored and in close contact with healthcare professionals, any adverse effects will be registered early, and women will receive proper care.

### Ethics and dissemination

This study has received ethical permission from the Swedish Ethical Review Authority (Ref no 2021-06627-01; 2022-03842-02; 2023-05911-02). All participants will provide written informed consent on enrolling in the study through a digital secure identification system. The findings of the study will be disseminated to the scientific community through peer-reviewed publications (applying relevant author guidelines for coauthorship) and conferences and to end-users, healthcare professionals and other stakeholders via traditional and social media, as well as relevant professional conferences and other specially organised seminars or meetings. No professional writers will be used.

## supplementary material

10.1136/bmjopen-2024-089355 online supplemental file 1
