# Supplementary material for: SPARK: an mHealth intervention for self-management and treatment of gestational diabetes mellitus in Sweden – protocol for a randomised controlled trial
Source: BMJ Open. 2025 Mar 3;15(3):e089355. doi: 10.1136/bmjopen-2024-089355 (PMC11877236; doi:10.1136/bmjopen-2024-089355)
Supplement: online supplemental file 1 [file bmjopen-15-3-s001.docx]

#
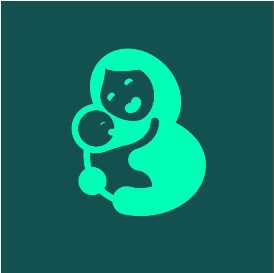
Participant consent form for the SPARK randomised controlled trial

**Etikprövningsmyndigheten 2023-05911-02-460162**

**2023-10-02**

**Informed consent to participate in the trial**

I have received full oral and written information about the study and have had the opportunity to ask questions. I have received a copy of the written information that I can keep.

• I agree to participate in the SPARK randomised controlled trial

• I agree to my blood samples being saved in a biobank in the manner described in the written participant information about the trial.

| Place and date | Signature |
| --- | --- |
|  |  |
|  | Name |
|  |  |

**Informed consent to participate in future research**

I have received information that the blood samples that I have provided may be relevant for future research that is not described in the current written information about the SPARK trial that I have received. I have also received information that if my blood samples are to be used in future research, the Ethics Review Authority must conduct a new evaluation of the new project and decide in its evaluation whether I should be contacted again for new informed consent.

• I agree that my blood samples may be saved for future research.

| Place and date | Signatures |
| --- | --- |
|  |  |
|  | Name |
|  |  |
