# Supplementary material for: SPARK: an mHealth intervention for self-management and treatment of gestational diabetes mellitus in Sweden – protocol for a randomised controlled trial
Source: BMJ Open. 2025 Mar 3;15(3):e089355. doi: 10.1136/bmjopen-2024-089355 (PMC11877236; doi:10.1136/bmjopen-2024-089355)
Supplement: Supplementary file 2 [file bmjopen-2024-089355.reviewer_comments.pdf]

PEER REVIEW HISTORY

BMJ Open publishes all reviews undertaken for accepted manuscripts. Reviewers are asked to complete a checklist review form and are provided with free text boxes to elaborate on their assessment. These free text comments are reproduced below.

ARTICLE DETAILS

Title (Provisional)

SPARK- an mHealth intervention for self-management and treatment of gestational diabetes mellitus in Sweden: protocol for a randomised controlled trial

Authors

Lilliecreutz, Caroline; Söderström, E; Ersson, Matilda; Bendtsen, Marcus; Brown, Victoria; Kaegi-Braun, Nina; Linder, Rebecka; Maddison, R; Chisalita, Simona; Lof, Marie

---

VERSION 1 - REVIEW

**Reviewer**	**1**
**Name**	**Borgen, Iren**
**Affiliation**	**VID Specialized University, Faculty of Health Sciences**
**Date**	**06-Sep-2024**
**COI**	**No**

Thank you for the opportunity to review the manuscript "SPARK- an mHealth intervention for self-management and treatment of gestational diabetes mellitus: protocol for a randomised controlled trial". It is an interesting and important topic.

I have the following comments to the manuscript:

1. The women in the intervention group will download the SPARK

app. Where will the data (blood glucose values) from the women be stored? In her app or in a cloud service?

2. Please consider to include more information about the Glooko app for the standard care group (e.g. data storage).

3. Please consider to include information for adverse outcomes in the manuscript. The women will be under close contact with health care professionals therefore any harm may be detected at an early stage and proper care will be given.

All the best,

Iren Borgen

Associate professor

**Reviewer**	**2**
**Name**	**Garnweidner-Holme, Lisa**
**Affiliation**	**Oslo Metropolitan University Kjeller Campus**
**Date**	**13-Nov-2024**
**COI**	**I have no competing interests**

Strengths and limitations of the study: The author should clarify why this study is an effectiveness trial

The authors write that "There may be some women who do not speak any of the four languages and thus cannot be enrolled, however, this limitation is minor as the main languages in this population are covered." – the authors can elaborate to include illiterate women as well

Introduction, page 7, lines 30-60: The introduction part can be improved to present how women with GDM are followed-up in the country/site where the study will be conducted vs globally/other countries.

The introduction could also benefit by including studies about how women with GDM/their health professionals experience mHealth tools; eg.: Helmersen M, Sørensen M, Lukasse M, Laine HK, Garnweidner-Holme L. Women's experience with receiving advice on diet and Self-Monitoring of blood glucose for gestational diabetes mellitus: a qualitative study. Scand J Prim Health Care. 2021; Garnweidner-Holme L, Hoel Andersen T, Sando MW, Noll J, Lukasse M. Health Care Professionals' Attitudes Toward, and Experiences of Using, a Culture-Sensitive Smartphone App for Women with Gestational Diabetes Mellitus: Qualitative Study. JMIR Mhealth Uhealth. 2018

Methods:

«satisfactory levels» of blood glucose should be described;

Inclusion and exclusion criteria: Basic language/reading skills could also be an inclusion criteria;

**Reviewer**	**3**
**Name**	**Roesler, Anna**
**Affiliation**	**CSIRO**
**Date**	**16-Dec-2024**

COI **nil**

The paper reads well.

There are some grammatical edits required throughout (e.g. pg 8 line 38 ... work, including, ... and line 52 behaviours, based ... pg 9 line 33 'in' line 35 Support (no need to capitalise the S)

VERSION 1 - AUTHOR RESPONSE

Reviewer 1 (Mrs. Iren Borgen, VID Specialized University)
Thank you for the opportunity to review the manuscript "SPARK- an mHealth intervention for self-management and treatment of gestational diabetes mellitus: protocol for a randomised controlled trial". It is an interesting and important topic.

Response: Thank you for the kind words about our manuscript.

1. The women in the intervention group will download the SPARK app. Where will the data (blood glucose values) from the women be stored? In her app or in a cloud service?

Response: Thank you – this is a very relevant comment. When a woman has taken her blood glucose using a glucose meter, the value is transferred via Bluetooth to the SPARK app. The woman is asked to press a button called "Save" and data is then transferred to a cloud service located in Sweden and at the same time showed in the SPARK app and in the health care professionals user interface. No data is stored on the woman's smartphone, so every time she uses the app, the data is collected from the cloud service. The procedure was approved by health care before study initiation and follows current legislations for our country. This information has now been added to the manuscript (page 10, lines 18-24).

2. Please consider to include more information about the Glooko app for the standard care group (e.g. data storage).

Response: Thank you –the Glooko app is a cloud service which has been approved for and used for standard diabetes care in Sweden. It is only used to upload glucose levels for women in the control group. We have included this requested information in the revised manuscript (page 11, lines 9-11).

3. Please consider to include information for adverse outcomes in the manuscript. The women will be under close contact with health care professionals therefore any harm may be detected at an early stage and proper care will be given.

Response: Thank you- good point. We consider the risk of potential adverse events with the intervention as minimal as the SPARK platform was developed in close collaboration with health care professionals working with GDM treatment (obstetricians as well as endocrinologists), and in accordance with current clinical guidelines. The women are carefully monitored and in close contact with the health care professionals and, if any adverse events still should occur, they will be detected early, and women will be provided proper care. We added as section with this information (page 21, lines 13-19).

Reviewer 2 (Prof. Lisa Garnweidner-Holme, Oslo Metropolitan University Kjeller Campus)
1.Strengths and limitations of the study: The author should clarify why this study is an effectiveness trial
Response: Thank you -good point. We have rephrased this sentence and removed "effectiveness trial" in this section (revised manuscript, page 4), and we have also revised a similar statement in the discussion (revised manuscript, page 22, line 22).

2.The authors write that "There may be some women who do not speak any of the four

languages and thus cannot be enrolled, however, this limitation is minor as the main languages in this population are covered." – the authors can elaborate to include illiterate women as well.
Response: We have clarified this sentence as suggested by the reviewer in the revised manuscript (page 4).

3. Introduction, page 7, lines 30-60: The introduction part can be improved to present how women with GDM are followed-up in the country/site where the study will be conducted vs globally/other countries.
Response: Thank you- good point. Women with GDM in Sweden are followed-up in accordance with international guidelines with the primary treatment being dietary and physical activity modifications and blood glucose monitoring followed by pharmaceutical treatment (metformin and thereafter insulin if required). In other words, GDM care is similar to many other countries, however, how the primary and secondary care is organised and executed may differ which motivates that mHealth interventions are developed for and evaluated in different health care settings to enable implementation at scale. This has been clarified in the revised version of the manuscript (page 6, line 18-23).

4. The introduction could also benefit by including studies about how women with GDM/their health professionals experience mHealth tools; eg.: Helmersen M, Sørensen M, Lukasse M, Laine HK, Garnweidner-Holme L. Women's experience with receiving advice on diet and Self-Monitoring of blood glucose for gestational diabetes mellitus: a qualitative study. Scand J Prim Health Care. 2021; Garnweidner-Holme L, Hoel Andersen T, Sando MW, Noll J, Lukasse M. Health Care Professionals' Attitudes Toward, and Experiences of Using, a Culture-Sensitive Smartphone App for Women with Gestational Diabetes Mellitus: Qualitative Study. JMIR Mhealth Uhealth. 2018
Response: Thank you- good point. We have added these articles as well as another article to the introduction to expand on women with GDM and health care professionals' views and experiences on mHealth tools (revised manuscript, page 5 lines 20-22 and page 6 lines 16-17; references 17, 28-29).

5. Methods: «satisfactory levels» of blood glucose should be described;
Response: Thank you- good point- this was somewhat unclear in our first version of the manuscript. We have clarified "satisfactory levels" in the revised manuscript (page 10, lines 3-6 and page 11, lines 8-9).

6. Inclusion and exclusion criteria: Basic language/reading skills could also be an inclusion Criteria'.
Response: Thank you. We have clarified the inclusion criteria as suggested by the reviewer (revised manuscript, page 11, lines 17-19).

Reviewer 3 Dr. Anna Roesler, CSIRO
The paper reads well.
There are some grammatical edits required throughout (e.g. pg 8 line 38 ... work, including, ... and line 52 behaviours, based ... pg 9 line 33 'in' line 35 Support (no need to capitalise the S)
Response: Thank you. We have corrected these grammatical errors except for the capitalisation of S in Support as this is the first word of a sentence and should be capitalised. The revised manuscript has also been reviewed for grammar by a professional proofreader company with minor edits been done accordingly throughout the manuscript (also marked as tracked changes).
